# Controlled release of hydrogel-encapsulated mesenchymal stem cells-conditioned medium promotes functional liver regeneration after hepatectomy in metabolic dysfunction-associated steatotic liver disease

**DOI:** 10.1186/s13287-024-03993-w

**Published:** 2024-11-04

**Authors:** Naoya Kasahara, Takumi Teratani, Junshi Doi, Shinichiro Yokota, Kentaro Shimodaira, Yuki Kaneko, Hideyuki Ohzawa, Yasunaru Sakuma, Hideki Sasanuma, Yasuhiro Fujimoto, Taizen Urahashi, Hideyuki Yoshitomi, Hironori Yamaguchi, Joji Kitayama, Naohiro Sata

**Affiliations:** 1 Department of Surgery, Jichi Medical University, Shimotsuke, Japan; 2 Division of Translational Research, Jichi Medical University, Shimotsuke, Japan; 3 Department of Surgery, Japanese Red Cross Otsu Hospital, Otsu, Japan; 4 Department of Transplant Surgery, Nagoya University Hospital, Nagoya, Japan; 5 Department of Surgery, Dokkyo Medical University Saitama Medical Center, Koshigaya, Japan

**Keywords:** Non-alcoholic fatty liver disease, Hepatectomy, Liver regeneration, Adipose tissue-derived mesenchymal stem cells, Conditioned medium, Hydrogel, Controlled release, Regenerative medicine

## Abstract

**Background:**

Globally, prevalence of metabolic dysfunction-associated steatotic liver disease (MASLD) is increasing, and there is an urgent need to develop innovative therapies that promote liver regeneration following hepatectomy for this disease. Surgical excision is a key therapeutic approach with curative potential for liver tumors. However, hepatic steatosis can lead to delayed liver regeneration and higher post-operative complication risk. Mesenchymal stem cells-conditioned medium (MSC-CM) is considered a rich source of paracrine factors that can repair tissues and restore function of damaged organs. Meanwhile, hydrogels have been widely recognized to load MSC secretome and achieve sustained release. This study aimed to evaluate the therapeutic effect of hydrogel-encapsulated MSC-CM on liver regeneration following partial hepatectomy (PHx) in a rodent model of diet-induced hepatic steatosis.

**Methods:**

Male Lewis rats were fed with a methionine and choline–deficient diet. After 3 weeks of feeding, PHx was performed and rats were randomly allocated into two groups that received hydrogel-encapsulated MSC-CM or vehicle via the intra-mesenteric space of the superior mesenteric vein (SMV).

**Results:**

The regeneration of the remnant liver at 30 and 168 h after PHx was significantly accelerated, and the expressions of proliferating cell nuclear antigen were significantly enhanced in the MSC-CM group. MSC-CM treatment significantly increased hepatic ATP and β-hydroxybutyrate content at 168 h after PHx, indicating that MSC-CM fosters regeneration not only in volume but also in functionality. The number of each TUNEL- and cleaved caspase-3 positive nuclei in hepatocytes at 9 h after PHx were significantly decreased in the MSC-CM group, suggesting that MSC-CM suppressed apoptosis. MSC-CM increased serum immunoregulatory cytokine interleukin-10 and interleukin-13 at 30 h after PHx. Additionally, mitotic figures and cyclin D1 expression decreased and hepatocyte size increased in the MSC-CM group, implying that this mode of regeneration was mainly through cell hypertrophy rather than cell division.

**Conclusions:**

MSC-CM represents a novel therapeutic approach for patients with MASLD requiring PHx.

**Supplementary Information:**

The online version contains supplementary material available at 10.1186/s13287-024-03993-w.

## Background

Globally, metabolic dysfunction-associated steatotic liver disease (MASLD) constitutes 25% to 45% of chronic liver diseases [1], and its estimated prevalence worldwide increased to over 30% in 2019 reflecting the escalating obesity epidemic [2]. MASLD is a major contributing factor to chronic liver disorders. It consists of metabolic dysfunction-associated steatohepatitis (MASH) and metabolic dysfunction-associated steatotic liver (MASL). Conditions are characterized by lobular inflammation, hepatocyte ballooning, varying levels of pericellular fibrosis, and potential development of hepatocellular carcinoma [3].

MASLD is marked by a persistent injury to the parenchyma, involving repetitive phases of damage and recovery. Therefore, surgical excision remains one of the most potent curative therapeutic approaches for liver tumors; however, there is an increased risk of liver failure when hepatic steatosis is present [4]. Liver steatosis hinders ability to regenerate and compensate for lost volume after hepatectomy [5]. Some studies have suggested that presence of steatosis in patients prior to liver resection can lead to a growing risk of liver failure and postoperative complications following hepatectomy, which restricts surgical options in patients with fatty liver [6]. Thus, novel therapeutic means are needed to trigger a controlled regenerative response of the liver in this pathological condition.

Recent studies have shown that mitochondrial dysfunction is a key contributor to the initiation and progression of MASLD [7]. According to prior research, mitochondrial fatty acid oxidation (FAO) capacity and rate-limiting enzyme function in β-oxidation were reduced by roughly 40–50% in MASH patients compared to healthy individuals [8]. The mitochondrion plays a vital role in generating adenosine triphosphate (ATP) through oxidative phosphorylation and β-oxidation regulation, as well as producing ROS (reactive oxygen species) that cause cellular damage. Mitochondrial dysfunction leads to impaired ATP production, resulting in cellular functional impairment. Additionally, damaged mitochondria produce excessive ROS and thereby induce a negative spiral known as a “vicious cycle” [9]. Therefore, improving mitochondrial function is a promising approach for treatment of MASLD.

Mesenchymal stem cells (MSCs) demonstrate high proliferative capacity and self-renewal ability. These secrete various growth factors to induce proliferation and differentiation of organ-constituent cells at the site of injury, thereby achieving organ function restoration [10]. MSCs also contribute to reducing inflammation by secreting various immunosuppressive and immune-regulatory factors [11]. Thus, due to their paracrine action and immunoregulatory function, MSCs have become a promising resource for cell transplantation. However, MSCs transplantation has limitations including low survival rate in vivo, depleted regenerative potential, and post-transplantation reduced cell differentiation.

It has been suggested that MSCs primarily exert their therapeutic effects through the secretion of trophic factors [12]. The MSCs culture media comprising the secretome is referred as MSCs conditioned medium (MSC-CM); it is considered a rich source of paracrine factors such as growth factors and cytokines [13]. Cell-free MSC-CM therapy confers key advantages over stem-cell based applications for important reasons. First, if administered intravascularly MSC-CM mitigates concerns with transplantation of living cells such as immune compatibility, tumorigenicity, and emboli formation. Second, MSC-CM is available in large-scale production and storage, so off-the-shelf therapy is feasible for treatment of acute clinical conditions. For these reasons, cell-free MSC-CM is a potential alternative to MSCs transplantation.

Meanwhile, hydrogels have received much attention in regenerative medicine, especially as a candidate drug delivery vehicle. Multiple reports have shown that hydrogel-encapsulated MSC-CM can continuously release growth factors and cytokines, which exerts pro-regenerative effects [14]. Although few studies have reported the intra-mesenteric space as an administration route for test drugs, systemic and splanchnic responses to intra-mesenteric and intravenous test drug were reported to be similar [15].

Some studies have demonstrated that MSCs transplantation and MSC-CM administration alleviate pathological changes in MASLD animal models [16, 17]. However, few studies have investigated effects of MSC-CM on regeneration after liver resection in MASLD. The aim of this study is to verify the therapeutic effect of MSC-CM on liver regeneration and explore its role in lipid metabolism after partial hepatectomy. To optimize utility of both MSC-CM and hydrogels, we administered MSC-CM encapsulated in hydrogel in the intra-mesenteric space of the superior mesenteric vein (SMV) to support sustained delivery to remnant liver though the portal vein.

## Methods

### Animals

Six- to seven-week-old male Lewis rats were purchased from Charles River (Breeding Laboratories, Kanagawa, Japan). Rats were fed standard chow (CE-2; CLEA Japan Co., Ltd., Tokyo, Japan). All animals were maintained in a specific pathogen free animal facility at Jichi Medical University under a temperature- and humidity-controlled environment with alternating 12-h light/dark cycles with free access to food and water ad libitum. Animals received humane care which met the institutional guidelines for animal welfare. All experiments were conducted with approval from the Animal Care Committee of Jichi Medical University (approval number: 15–214) and performed in accordance with ARRIVE guidelines and the Japanese Guidelines for Animal Research.

### Preparation of AT (adipose tissue derived) -MSC conditioned medium

The established method of rat AT-MSCs was described previously [18]. In brief, adipose tissue obtained from the inguinal region of wild-type LEW rat was minced into pieces. After shaking with an equal volume of PBS, the mixture was separated into two phases. The upper phase was digested with 0.075% collagenase (type 1) (Wako, Tokyo, Japan) in PBS for 1.5 h at 37 °C with shaking. The digested tissue was mixed with minimum essential medium α modification (MEMα; Gibco-BRL, Tokyo, Japan), added with 10% fetal bovine serum (FBS; Gibco), and incubated for 10 min at room temperature. Subsequently, the lower phase was centrifuged and the resulting isolated AT-MSCs were seeded onto 100-mm tissue culture dishes (Thermo Scientific, Tokyo, Japan) cultured in MEMα supplemented with 10% FBS. AT-MSCs were cultured to reach confluence followed by a wash with PBS [–] and incubation in serum-free MEMα medium (GIBCO). After 2 days of culture, the supernatant was collected and then centrifuged, filtered, and concentrated at 12,000 rpm using Amicon Ultra centrifugal filters (Millipore, Tokyo, Japan; molecular weights 3, 10, 30, 50, and 100 kDa). Liquids concentrated 1000-fold for each fraction's molecular weight were mixed to be utilized as MSC-CM in this study.

### Characterization of rat AT-MSCs

The AT-MSCs at passage 5 were incubated with 10 μL of Fc-blocker for 5 min and immunostained with Abs for 30 m at room temperature. After washing and labeling of dead cells with 7-AAD (Invitrogen), cells were analyzed using a FACSCalibur and CellQuest Pro Software (Becton Dickinson). AT-MSCs were stained with antibodies to CD29 (#102,207, BioLegend, San Diego, CA) CD31(#FAB3628G, R&D Systems, Minneapolis, MN), CD34 (sc-7324 AF647, Santa Cruz Biotechnology, Santa Cruz, CA), CD90 (#567,361, BioLegend) and CD105 (10,862–1-AP, Proteintech, Rosemont, IL) and their expressions were examined using FACSCalibur.

### Trilineage differentiation of AT-MSCs

The differentiation potential of MSCs (passage 3) into adipocytes, osteocytes, or chondrocytes was evaluated using differentiation-induction media (PT-3002, PT-3003, PT-3004; Lonza, Basel, Switzerland) according to the manufacturer’s protocols as previously described. After the specific differentiation culture, cells were analyzed by cytochemical staining with Oil Red-O, Alizarin Red, or Safranin-O, respectively.

### Experimental protocol

**Fig. 1:**
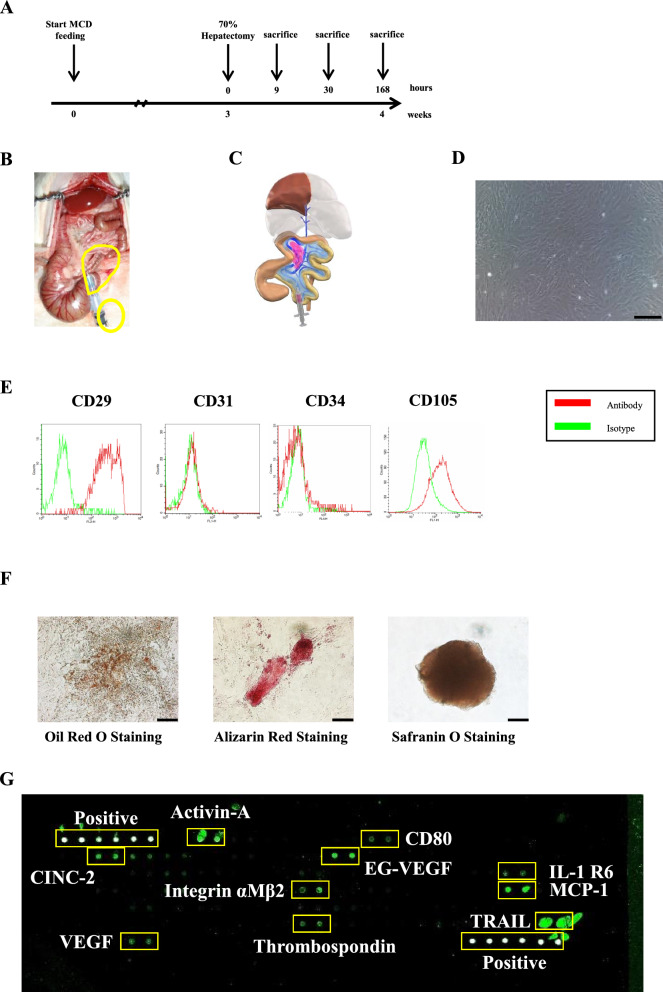
Experimental protocol, characterization and tri-lineage differentiation of AT-MSCs and cytokine array analysis of MSC-CM. **A** After 3 weeks of MCD diet feeding, hepatectomy was performed. Rats were sacrificed at 9 h, 30 h and 168 h after hepatectomy. **B** Our model of 70% hepatectomy. **C** In the MSC-CM group, MSC-CM with peptide hydrogel as delivery vehicle was administered into the intra-mesenteric space of ileo-colic vein perfused area. In the control group, peptide hydrogel alone was administered. **D** Phase contrast images of rat AT-MSCs (original magnification, × 100, scale bars represent 200 μm). **E** Detection of rat AT-MSCs surface antigens by flow cytometry. AT-MSCs at passage 3–5 were stained with mAbs to CD29, CD31, CD34, CD105 and their expressions were examined. **F** AT-MSCs at passage 3 were induced to differentiate into adipocytes, osteoblasts, and chondrocytes-like cells (original magnification, × 200, scale bars represent 100 μm). **G** Images of cytokine array blots probed with MSC-CM used in this study

We started feeding rats with a methionine and choline-deficient (MCD) diet (F2MCD; Oriental Yeast Co., Ltd., Tokyo, Japan) from 8 weeks old until the termination of experiment. After a MCD diet for 3 weeks, partial hepatectomy (PHx) was performed. During the surgical procedure, rats were allocated randomly into 2 groups: MSC-CM administered group and Control group (Fig. 1A). Animals were euthanized with an inhalation of excess isoflurane after the hepatectomy of 9 h, 30 h and 168 h. Blood samples were collected from the inferior vena cava for liver function tests and cytokine array analysis, and the liver was harvested and weighed. Standard diet-fed 11-week-old rats, and 11-week-old rats that had been fed an MCD diet for 3 weeks starting from 8 weeks of age were euthanized to harvest liver tissues as specimens. Liver tissue samples were suitably stored for subsequent procedures.

### Surgical procedure

Animals were fasted overnight and all procedures were performed under inhalation anesthesia using 1.5% isoflurane. After midventral laparotomy, the left lateral lobe, left portion of the medial lobe, right inferior superior lobes, and caudate lobes were resected with ligation of respective narrow pedicle using 4–0 silk, leaving only the medial lobe behind (Fig. 1B, C) as described previously [19]. Immediately after the hepatectomy, 1.0 ml of PuraMatrix™ (354,250, Corning, Corning, NY) was infused into the intra-mesenterium space of SMV perfused area with a 23-gauge needle followed by additional infusion of 100 μl of phosphate-buffered saline (PBS) or MSC-CM into the antecedently infused PuraMatrix™ (Fig. 1B, C), by which sustained release of MSC-CM to the liver via portal vein was feasible. The abdomen was closed with 4–0 nylon running sutures, and the rats were allowed to awaken from anesthesia.

### Assessment of cytokines in MSC-CM

The cytokines in MSC-CM were measured using a biotin label-based rat antibody array (AAR-BLG and QAR-INF-1–1, Ray-Biotech, Norcross, GA). In brief, MSC-CM was dialyzed with dialysis buffer, labeled with biotin and incubated with arrays overnight at 4 °C. Glass slides were then incubated with Cy3-conjugated streptavidin overnight at 4 °C. Finally, the arrays were scanned and analyzed.

### Assessment of liver function and serum cytokines

Blood samples were centrifuged for 10 min at 3000 rpm, and serum was collected. Serum alanine aminotransferase (ALT) was measured as an indicator of degrees of hepatocellular damage using a FUJIFILM DRI-CHEM 3500 machine (FujiFilm, Tokyo, Japan) and FUJI DRY CHEM SLIDES (FujiFilm) for GPT/alanine transaminase (ALT)-PIII. To assess the serum cytokines and chemokines, multiplexed sandwich ELISA-based quantitative array (QAR-CYT-3, Ray-Biotech) was used according to the manufacturer’s directions (n = 4 in each group at 9 h and 30 h after PHx). The arrays were scanned with GenePix® 4400A Microarray Scanner (Molecular Devices, Sunnyvale, CA), and images were analyzed using RayBio analysis tool.

### Gene expression profiling

RNA was extracted from liver samples of rats 9 h after PHx, standard diet-fed rats without PHx (SD Hpx(-)), and MCD diet-fed rats without PHx (MCDD Hpx(-)) (n = 4/group). Total RNA was extracted from frozen liver tissues using RNeasy Lipid Tissue Mini Kit (Qiagen) according to the manufacturer’s instructions. The sense-strand DNA was amplified and then biotinylated according to the manufacturer’s protocol from 250 ng total RNA (Expression Analysis WT Plus Technical Manual 2013; Affymetrix, Santa Clara, CA). Following fragmentation, the biotinylated sense-strand DNA underwent a 16-h hybridization at 45 °C on the GeneChip Rat Gene 2.0 ST Array. The GeneChips were washed and stained at the Affymetrix Fluidic Station 450, followed by scanning with the Affymetrix Gene Chip Scanner 3000 7G System. Affymetrix tools were used for preprocessing, and two algorithms designed to summarize microarray probes (robust multichip average algorithm and probe logarithmic intensity error) were implemented to enhance the statistical power in analyzing gene differentiation. For each probe set, gene and gene ontology annotations were established based on the annotation files available on the Affymetrix website (http://www.affymetrix.com/). Following thresholding and filtering, the log2-normalized expression values were analyzed using a Database for Annotation, Visualization and Integrated Discovery (DAVID) and a comprehensive gene ontology (GO) term analysis.

Fisher’s exact test was used to calculate a p-value determining the probability that the biological function assigned to that data set is explained by chance alone. The predicted activation state and activation z-scores were computed based on the direction of fold change values observed in the gene expression data.

### Histological analysis

Liver tissues were fixed in 10% formalin for more than 24 h. Paraffin-embedded liver tissue Sects. (4 μm) were subjected to hematoxylin and eosin staining for histological analysis. Hepatocyte number per high-power field magnification (HPF 400X) was counted in 10 randomly selected fields per liver.

### Immunohistochemical staining

After deparaffinization and rehydration, slides were treated with 3% hydrogen peroxide. Sections were subjected to antigen retrieval by heating to boiling in a 0.1 M Citrate Buffer Antigen Retrieval Solution (pH 6.0) for 20 min. To prevent non-specific binding of antibodies, sections were treated with 15% goat serum for 30 m. For immunohistochemical staining of PCNA, Ki-67, Cyclin D1, cleaved Caspase-3, and CD31, slides were then incubated with anti-PCNA antibody (sc-7907, Santa Cruz Biotechnology, Santa Cruz, CA), anti-Ki67 antibody (E1870, Spring Bioscience, Pleasanton, CA), anti-cyclin D1 antibody (sc-753, Santa Cruz Biotechnology), anti-cleaved Caspase-3 antibody (#9664, Cell Signaling Technology, Danvers, MA), anti-CD31 antibody (ab182981, Abcam, Cambridge, UK), anti-CD163 antibody (sc-58965, Santa Cruz Biotechnology), and anti-E-cadherin antibody (20,874-1-AP, Proteintech) at 4℃ overnight. The signal was detected with the ABC kit and DAB kit (Vector Laboratories, Burlingame, CA). Sections were counter-stained with hematoxylin. In morphometrical quantification of the staining area using Fiji/ImageJ (National Institutes of Health), all acquisition settings were the same for sections of both group within each immunostaining. Images were thresholded and quantified in Fiji/ImageJ software. The threshold within each set was kept constant for all images of both groups within a given confocal channel. Histological slides of the livers were analyzed in a blinded manner.

### Identification of apoptotic hepatocytes

Three randomly selected HPF (200X) were selected to calculate the area stained by cleaved Caspase-3. The staining area was morphometrically quantified using Fiji/ImageJ (n = 4–5/group). TUNEL assay was performed on 4-μm liver sections using the FD Apop Kit (FD NeuroTechnologies, Columbia, MD) according to the manufacturer’s instructions. The number of TUNEL-positive cells was counted from 10 randomly selected HPF (400X) per slides (n = 4–5/group).

### Liver regeneration rate

To estimate regeneration rate after partial hepatectomy, resected livers were weighed at time of procedure. The remnant liver was excised and weighed when the rats were sacrificed. **L**iver regeneration rates were calculated as the ratio of remnant liver weight/estimated whole liver weight. The original whole liver weight was extrapolated by calculating the resected liver weight/0.663, based on previous results [15].

### Detection of proliferating hepatocytes

The number of PCNA-positive and Ki-67-positive cells per HPF was counted in 3 and 10 randomly selected fields per liver (n = 4–5/group). Three HPF (200X) were randomly selected for calculating the staining area of the Cyclin D1. Mitosis per HPF (200X) was counted at 5 randomly selected fields per liver of anti-Cyclin D1 stained slide.

### Measurement of hepatocyte size

Sections stained for E-cadherin were photographed at a magnification of 400X. The area of hepatocytes was measured by visualizing cell outlines through immunostaining with E-cadherin for 100 hepatocytes per rat using Fiji/ImageJ (n = 4–5/group).

### Evaluating damage to liver sinusoidal endothelial cells (SECs)

Evaluating damage to SECs was performed by immunohistochemical staining for CD31. To assess the stained area within the SECs, ten fields were selected randomly under high magnification (200X). The staining area was quantitatively analyzed using Fiji/ImageJ (n = 4–5/group).

### Morphometric Analysis of M2 macrophage populations

M2 Macrophage populations were analyzed on sections immunohistochemically stained for CD163 (M2 marker). Densities of M2 macrophages were analyzed on 5 fields at 200X　on one representative slide. The mean densities were expressed as the number of positive cells per mm^2^.

### Measurement of liver lipid content

The measurement of triglyceride, total cholesterol, free cholesterol and phospholipid levels in rat livers using Folch’s extraction procedure was outsourced to Immuno-Biological Laboratories Co., Ltd. (Fujioka, Japan).

### Glycogen periodic acid Schiff stain

For periodic acid Schiff (PAS) stain, liver specimens were subjected to three 5-min PBS washes and then exposed to periodic acid for 8 min. Following two distilled water washes, staining with Schiff reagent was carried out for 20 min. After three distilled water rinses, specimens were treated with hematoxylin and inspected under a microscope. The staining area was quantitatively analyzed using Adobe Photoshop (n = 4–5/group).

### Quantification of liver ATP, β-hydroxybutyrate and acetyl CoA content

The hepatic ATP, β-hydroxybutyrate and acetyl CoA concentration were measured using an ATP Assay Kit (ab83355, Abcam), β-Hydroxybutyrate (Ketone Body) Fluorometric Assay Kit (700,740, Cayman Chemical, Ann Arbor, MI) and Acetyl CoA Assay Kit (ab87546, Abcam) according to manufacturer's instructions.

### Statistical analysis

Prism software (GraphPad Software, Inc., La Jolla, CA) was used for statistical analyses. Data are presented as mean ± SD. Student’s *t*-test was used for comparison between groups, and *P*-values were used to quantify statistical significance (*P < 0.05; **P < 0.01; ***P < 0.001).

## Results

### The expression of the CD-surface markers in AT-MSCs

The AT-MSCs used in this study exhibited a fibroblastic morphology with a bipolar spindle shape (Fig. 1D). Flow cytometry immune profiling revealed positive expression for putative mesenchymal stem markers CD29 and CD105. The endothelial marker CD31 and hematopoietic stem marker CD34 showed negative antigenic reactivity (Fig. 1E).

### Tri-lineage differentiation capabilities of AT-MSCs

As functional MSCs, AT-MSCs should exhibit multi-lineage differentiation potential (adipogenic, osteogenic and chondrogenic) according to guideline recommendations of the International Society for Cellular Therapy (ISCT). We studied the tri-lineage differentiation potential of AT-MSCs. AT-MSCs demonstrated adipocyte, osteocyte and chondrocyte differentiation by Oil Red-O positive staining of lipid droplets, Alizarin Red staining of calcium deposits, and Safranin O staining of proteoglycan (Fig. 1F).

### MSC-CM contained several kinds of cytokines including Activin-A and IL-10

The alignment of cytokine arrays is shown in Supplementary Table S1. Scanned image of cytokine array blots after incubation with MSC-CM are shown in Fig. 1G. The content of cytokines in MSC-CM were measured in a semiquantitative manner: Blots for TRAIL and Activin-A showed high signal intensity; MCP-1 and EG-VEGF showed moderate signal intensity; and CINC-2, Integrin αMβ2, VEGF, Thrombospondin, IL-1R6 and CD80 showed weak signal intensity. Next, content of inflammatory cytokines in MSC-CM were measured in quantitatively (Table 1).

**Table 1:** Content of inflammatory cytokines in MSC-CM used in the experiment

Cytokine	Concentration (pg/ml)
IFN-γ	ND
IL-1α	ND
IL-1β	ND
IL-2	2243.1
IL-4	18.0
IL-6	ND
IL-10	982.2
IL-13	ND
MCP-1	6396.7
TNFα	ND

*ND* not detected

### MSC-CM suppressed hepatectomy induced apoptosis

**Fig. 2:**
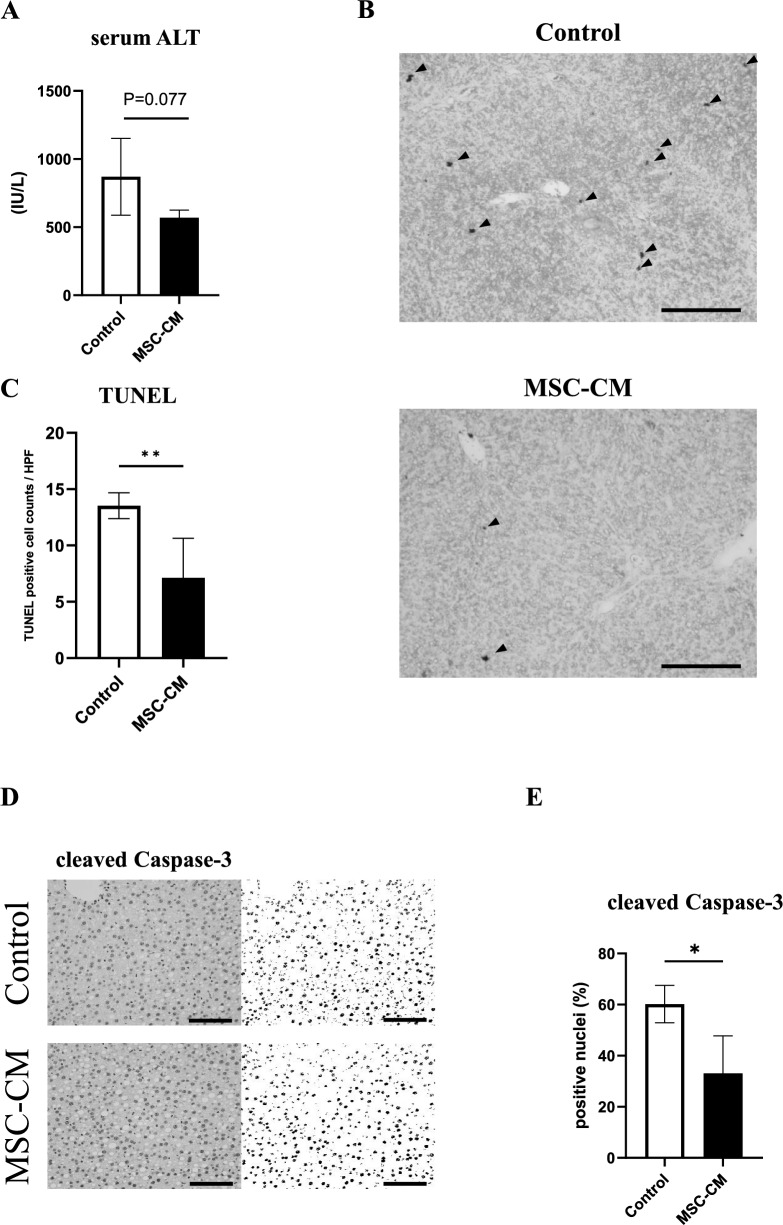
Assessments of apoptosis. **A** Serum ALT concentration at 9 h after hepatectomy. **B** Representative liver sections stained by TUNEL at 9 h after hepatectomy. Arrowheads indicate TUNEL positive nuclei of hepatocytes (original magnification, × 200, scale bars represent 200 μm). **C** Quantification of TUNEL-positive hepatocytes. **D** Quantification of cleaved Caspase-3 positive hepatocytes. **E** Representative liver sections stained by cleaved Caspase-3 at 9 h after hepatectomy (original magnification, × 200, scale bars represent 200 μm). Open bars, control group; closed bars, MSC-CM group. Data are presented as mean ± SD (*P < 0.05, n = 4–5/group)

The serum ALT levels of the MSC-CM group at 9 h after hepatectomy were lower compared with those of the control group, although not significantly different (*P* = 0.07, n = 4–5/group, Fig. 2A). To assess the effect of MSC-CM on apoptosis induced by hepatectomy, TUNEL analysis was performed on liver Sects. 9 h after hepatectomy. The number of apoptotic cells induced by hepatectomy in the MSC-CM group were significantly fewer compared with the control group (*P* < 0.01, n = 4–5/ group, Fig. 2B, C). Cleaved caspase-3 immunohistochemistry was performed and areas with relative cleaved caspase-3 positivity were examined to quantitatively evaluate the impact of MSC-CM on apoptosis. Relative cleaved caspase-3 positivity nuclei of MSC-CM group were significantly smaller than the control group (*P* < 0.01, n = 4–5/ group, Fig. 2D, E).

**Fig. 3:**
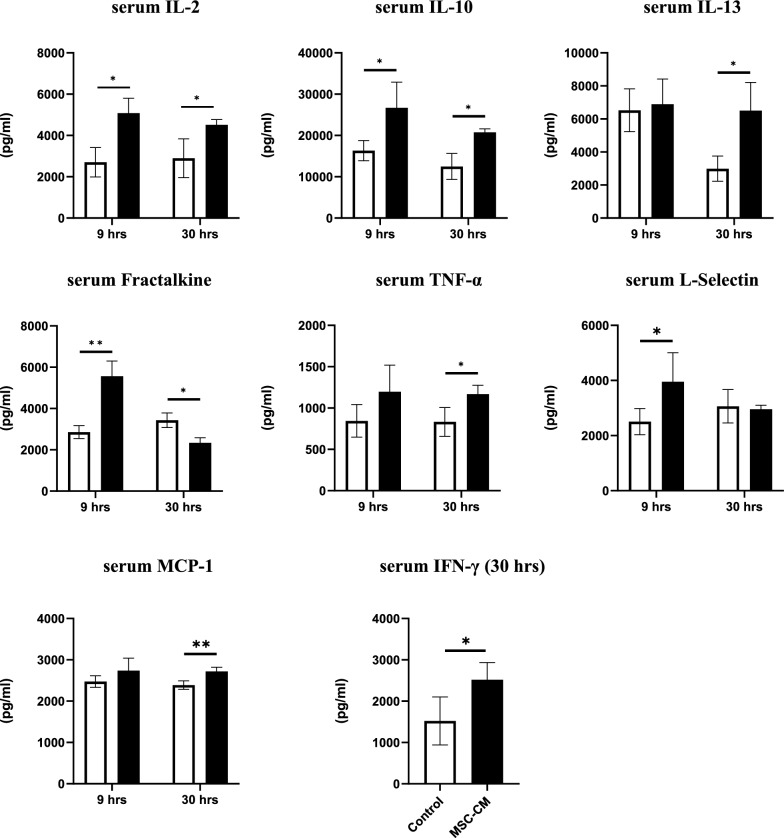
Serum concentrations of cytokines and chemokines (IL-2, IL-10, IL-13, Fractalkine, TNF-α, L-Selectin, MCP-1) at 9 h and 30 h after hepatectomy. The data of IFN-γ is shown only those of at 30 h after hepatectomy. Open bars, control group; closed bars, MSC-CM group. Data are presented as mean ± SD (*P < 0.05, **P < 0.01, n = 4/group)

Serum levels of cytokines and chemokines at 9 h and 30 h after hepatectomy are shown in Fig. 3. Analyzed mediators are interleukin (IL) -2, IL-10, IL-13, fractalkine, tumor necrosis factor α (TNF-α), L-selectin, monocyte chemotactic protein 1 (MCP-1) and interferon γ (IFN-γ). The levels of serum of IL-2 and IL-10 were significantly higher in the MSC-CM group compared with controls (*P* < 0.05, n = 4/group) at both 9 h and 30 h. The serum level of L-selectine was significantly higher in the MSC-CM group compared with controls (*P* < 0.05, n = 4/group) at 9 h and the level of serum of IL-13, TNF-α, MCP-1 and IFN-γ were significantly higher in the MSC-CM group compared with controls (*P* < 0.01 for MCP-1, *P* < 0.05 for others, n = 4/group) at 30 h. However, the level of serum fractalkine was significantly higher at 9 h and significantly lower in the MSC-CM group compared with controls (*P* < 0.01 for 9 h and *P* < 0.05 for 30 h, n = 4/group).

### MSC-CM promoted liver regeneration

**Fig. 4:**
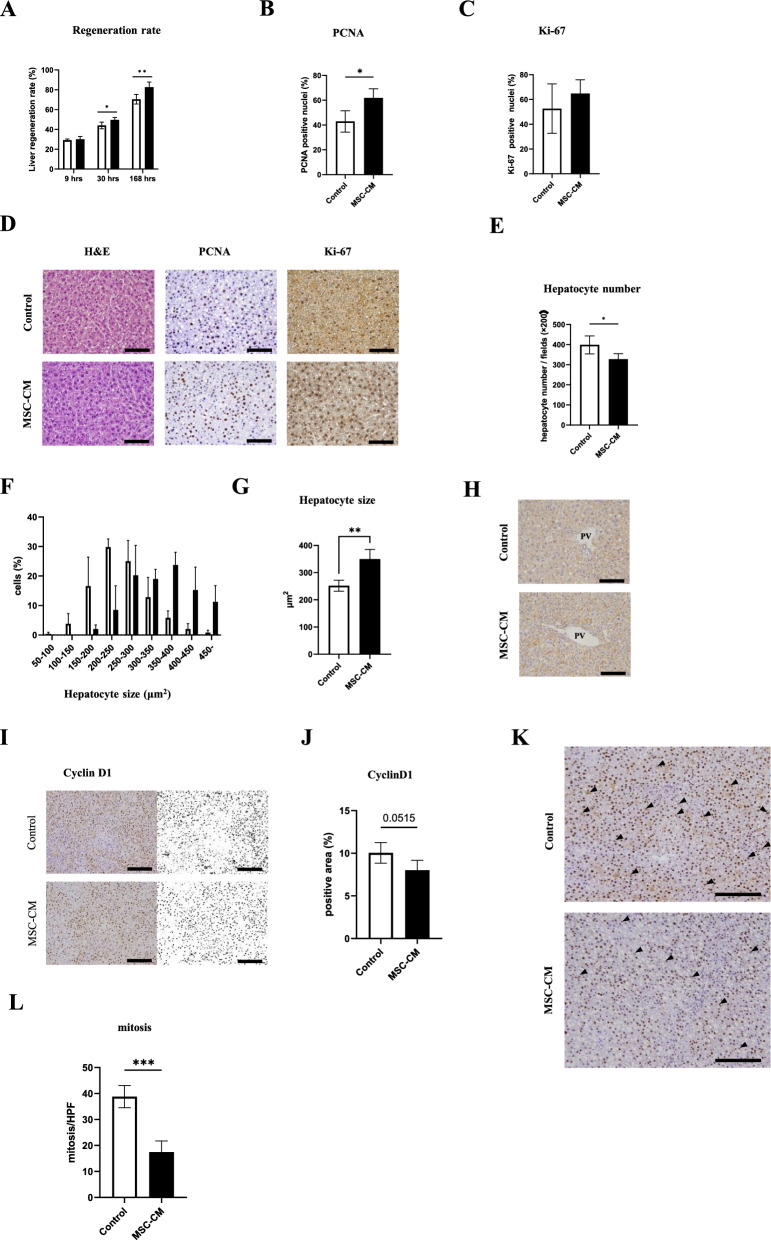
Assessments of liver regeneration. **A** Liver regeneration rates (%) at 9 h, 30 h, and 168 h after hepatectomy (9 h and 30 h: n = 4–5/group, 168 h: n = 5–8/group). **B** Quantification of PCNA-positive nuclei (%) at 30 h after hepatectomy (n = 4–5/group). **C** Quantification of Ki-67-positive nuclei in both groups at 30 h after hepatectomy (n = 4–5/group). **D** Representative immunohistochemical images from H&E, PCNA and Ki-67 at 30 h after hepatectomy (original magnification, × 400; scale bars represent 100 μm). **E** The number of hepatocytes per HPF in the liver sections at 30 h after hepatectomy (n = 4–5/group). **F** Hepatocyte size distribution. **G** Hepatocyte size in both groups. **H** Liver sections stained for E-cadherin are shown (original magnification, × 400, scale bars represent 100 μm). **I** Representative liver sections stained by Cyclin D1 at 30 h after hepatectomy (original magnification, × 400, scale bars represent 100 μm). **J** Relative Cyclin D1 positive area was morphologically quantified. **K** Representative liver sections stained by cyclin D1 at 30 h after hepatectomy. Arrows indicate mitotic figure in hepatocytes (original magnification, × 200, scale　bars represent 200 μm). **L** Quantification of mitotic figures in both groups. Open bars, control group; closed bars, MSC-CM group. Data are presented as mean ± SD (*P < 0.05; **P < 0.01; ***P < 0.001; n = 4–5/group)

All rats survived until sacrificed. The liver regeneration rates at 9 h, 30 h and 168 h after hepatectomy are shown in Fig. 4A. The liver regeneration rate was significantly higher in MSC-CM group at 30 h and 168 h compared to controls (at 30 h, regeneration rate of 49% vs. 44%, *P* < 0.05, n = 4–5/group; at 168 h, 82% vs. 70%, *P* < 0.01, n = 5–8/group). To investigate cell proliferation potential, PCNA expression which is a marker of cell cycle entry and is elevated in the nucleus during late G1 phase and S phase, was assessed by immunostaining. The rate of PCNA-positive cells in the MSC-CM group was significantly higher at 30 h after hepatectomy compared with controls (*P* < 0.05, n = 4–5/group, Fig. 4B, D). Next, Ki-67 immunostaining was performed. Unexpectedly, Ki-67 labeling index showed no significant difference between the groups (Fig. 4C, D).

Despite unaccompanied cell proliferation marker Ki-67 elevation, the liver regeneration rate was significantly higher at 30 h in the MSC-CM group. Thus, we hypothesized that cell hypertrophy rather than cell proliferation contributed to liver regeneration in this model. To assess whether hepatocytes were enlarged, we measured the number of hepatocyte nuclei at 30 h after PHx in each liver section. The number of nuclei per field was significantly decreased in the MSC-CM group compared to controls (*P* < 0.05, n = 4–5/group, Fig. 4E). Additionally, measurement of hepatocyte size revealed that hepatocyte size was significantly larger in the MSC-CM group versus controls (*P* < 0.01, n = 4–5/group, Figs. 4F–H).

To evaluate MSC-CM effects on cell cycle during liver regeneration, CyclinD1 immunohistochemistry was performed and areas with relative CyclinD1 positivity were examined. Compared to controls, the MSC-CM group tended to have smaller CyclinD1 positive staining areas, but the difference was not statistically significant (Fig. 4I, J). We also assessed the number of mitotic figures in the same sections of CyclinD1 stain. The number of mitotic figures in MSC-CM group was significantly smaller than controls (*P* < 0.001, n = 4–5/ group, Fig. 4K, L).

Taken together, we confirmed presence of MSC-CM-induced hypertrophic proliferation with reductive cell division after PHx of MASLD liver. To assess angiogenesis during liver regeneration, sinusoidal endothelial cell staining was performed with CD31 antibody in liver sections at 30 h post-surgery. The CD31 positive area percentage was comparable between the groups (Supplementary Fig. S1). Next, to explore involvement of M2 macrophages in liver regeneration, staining of CD163-expressing macrophages was performed, but CD163-positive cell numbers were not significantly different between groups (Supplementary Fig. S2).

### Changes in mRNA expressions in remnant liver by MSC-CM administration

To identify mechanisms by which MSC-CM promoted liver regeneration, we compared mRNA expressions at 9 h after PHx by microarray analysis. We hybridized 30,429 probe sets with amplified total RNA, filtering the raw data by removing entries not annotated with Symbol and Entrez ID, resulting in a total of 22,623 data points. We evaluated the differentially expressed genes (DEGs) by performing three comparisons as follows: (1) MCD diet without PHx versus standard diet without PHx to identify the genes affected by MCD diet (Supplementary Table S2); (2) MCD diet with PHx (control group) versus MCD diet without PHx to detect the genes affected by PHx (Supplementary Table S3); (3) MCD diet with PHx and MSC-CM (MSC-CM group) versus MCD diet with PHx (control group) (Table 2) to analyze genes affected by administration of MSC-CM.

**Table 2:** Differential gene expression analysis of control group versus MSC-CM group at 9 h. Top 10 DEGs in ascending order of FDRs

Symbol	logFC	logSIGNAL	*P* value	FDR
RGD1563861	2.52	10.6	6.28E−180	1.42E−175
RGD1311300.2	− 3.72	7.48	9.65E−63	1.09E−58
Rbm25l1	1.69	8.51	2.84E−38	2.14E−34
Rps24.1	2.76	7.00	2.97E−33	1.34E−29
Rps25	2.76	7.00	2.97E−33	1.34E−29
Timm23b	1.13	9.38	6.84E−29	2.58E−25
Cox7a2	− 3.84	6.13	2.32E−27	7.50E−24
Mir770	− 0.90	10.1	2.72E−25	7.69E−22
RGD1561195	− 1.82	7.54	3.08E−25	7.75E−22
LOC501421.6	− 2.16	6.92	1.00E−22	2.06E−19

*logFC* log_2_ (average expression level of MSC-CM group) − log_2_ (average expression level of control group), *logSIGNAL* ((log_2_ (average expression level of Group control group) + log_2_ (average expression level of MSC-CM group))/2, *P value* the p-value calculated by the exact test, the FDR (False Discovery Rate) calculated using the Benjamini–Hochberg method

**Fig. 5:**
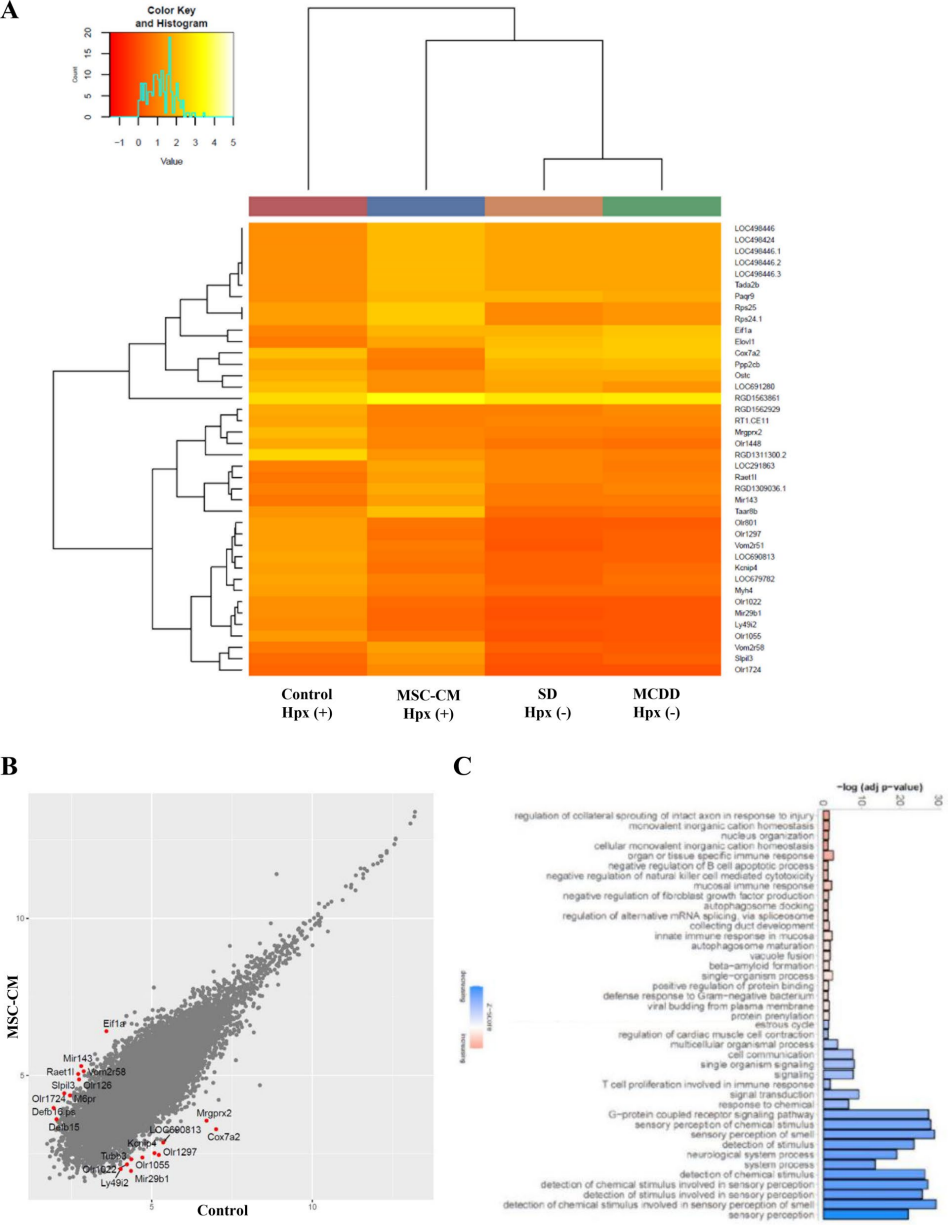
Differential expression analysis of liver tissue mRNA expression at 9 h for control group versus MSC-CM group. **A** Hierarchical clustering of four groups regarding the top 20 genes with the most significant fluctuations (standard diet-fed without PHx, MCD diet-fed without PHx, control group, MSC-CM group). **B** Scatter plots showing all the DEGs. **C** Bar chart of the most significant GO biological processes that are associated with up- and down-regulated genes. Red bars represent positive z-scores and blue bars indicate negative z-scores, with the color intensity reflecting the absolute magnitude of the z- score. X-axis represents the statistical significance of the enrichment (− log10(*p*-value))

Using the combination of an FDR < 0.05 and a fold change > 2 as thresholds for statistical significance, 509 genes were found to be up- or down-regulated in MSC-CM group compared with controls, and top 10 DEGs in ascending order of FDRs are shown in Table 2. *RPS24.1* is an essential ribosomal protein for ribosome small subunit 40S, and *Rps25* is a part of cytosolic small ribosomal subunit. *RGD1561195* is known to be 60S ribosomal protein L31-like. Notably, the expression of *Mir770* was enhanced in controls compared to MCD diet without PHx (Supplementary Table S3), while it showed a decrease in the MSC-CM group compared to controls (Table 2). Studies have shown that an increase in *Mir770* expression leads to worsening diabetic nephropathy via apoptosis [20], while reducing *Mir770* expression results in decreased apoptosis in glioma cell lines [21]. By using the respective top-20 most significantly up- and down-regulated genes, hierarchical clustering analysis was performed to assess correlations and expression profiles of the DEGs. In the Fig. 5A, the genes are colored red and yellow. Among the 40 genes mentioned above, olfactory receptors (ORs) such as *Olr1448*, *Olr801*, *Olr1297*, *Olr1022*, *Olr1055*, *Olr1724* are included.

Subsequently, a volcano plot graph (Supplementary Fig. S3) was created to visualize significant DEGs. The scatter plot graph (Fig. 5B) shows distribution of significantly expressed mRNAs. To analyze potential functions and biological pathways of the DEGs further, GO enrichment analyses in the biological process (BP) was performed for compared groups according to the *P*-value. The predicted activation state and computed activation z-score are based on the direction of fold change values in the gene expression data. The activation z-score infers probable states of activation, characterized as “increasing” or “decreasing”, and was considered significantly increased (or decreased) with an overlap p-value ≤ 0.05 and a z-score ≥ 2.0 (or ≤ -2.0) (Fig. 5C, Table 3). The down-regulated pathways included those related to G protein-coupled receptor signaling pathways, to detection of chemical stimulus involved in sensory perception of smell, and to sensory perception of smell and chemical stimulus, among others.

**Table 3:** GO Biological Processes that are enriched in DEGs in MSC-CM group compared with control group at 9 h. Top 10 GO biological processes in ascending order of *P* values

GO.ID	Term	Annotated	Significant	Expected	*P* value
GO:0007186	G protein-coupled receptor signaling pathway	981	155	107.11	9.00E−07
GO:0050911	Detection of chemical stimulus involved in sensory perception of smell	75	21	8.19	3.50E−05
GO:0002251	Organ or tissue specific immune response	45	14	4.91	0.0002
GO:0002227	Innate immune response in mucosa	31	11	3.38	0.00027
GO:0007608	Sensory perception of smell	110	25	12.01	0.00027
GO:0007606	Sensory perception of chemical stimulus	262	47	28.61	0.00041
GO:1,902,914	Regulation of protein polyubiquitination	28	10	3.06	0.00048
GO:1,902,916	Positive regulation of protein polyubiquitination	16	7	1.75	0.00086
GO:0019236	Response to pheromone	81	19	8.84	0.00095
GO:0002385	Mucosal immune response	41	12	4.48	0.00106

### MSC-CM affected lipid metabolism and improved energetic status in remnant liver

**Fig. 6:**
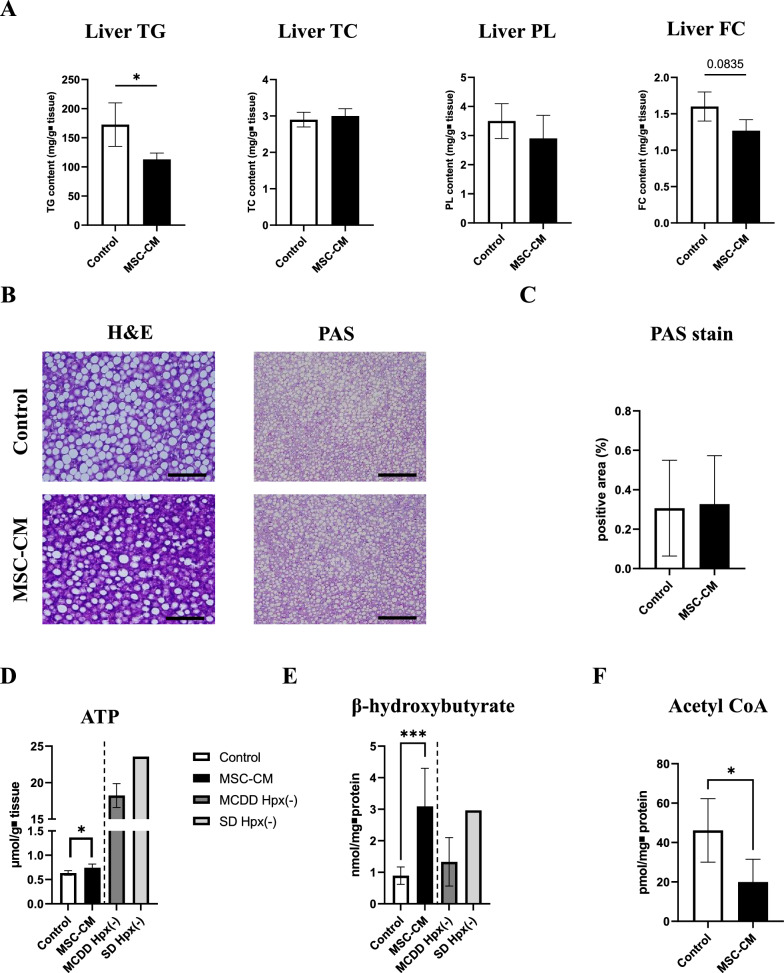
Effect of MSC-CM on lipid metabolism and functional recovery after hepatectomy. **A** Liver tissue triglyceride (TG), total cholesterol (TC), phospholipids (PL), and free cholesterol content in livers at 168 h after hepatectomy (n = 5–8/group). **B** Representative images of H&E (original magnification, × 400, scale bars = 100 μm) and PAS (× 200, scale bars = 200 μm) staining in liver slides at 168 h after hepatectomy. **C** Relative PAS staining positive area was morphologically quantified. **D** ATP concentrations in livers at 168 h after hepatectomy (n = 5–8/group). For reference, values for both the standard diet-fed group (SD Hpx(-)) and the MCD diet-fed group without PHx (MCDD Hpx(-)) were also shown.**E** β-hydroxybutyrate concentrations in livers at 168 h after hepatectomy (n = 5–8/group). Values for both the standard diet-fed group (SD Hpx(-)) and the MCD diet-fed group without PHx (MCDD Hpx(-)) were also shown. **F** Acetyl CoA concentrations in livers at 168 h after hepatectomy (n = 5–8/group). Open bars, control group; closed bars, MSC-CM group; dark gray bars, MCDD Hpx(-) group; light gray bars, SD Hpx(-) group. Data are presented as mean ± SD (*P < 0.05, ***P < 0.001, n = 5–8/group)

To evaluate effects of MSC-CM on lipid metabolism in liver regeneration, we measured hepatic lipid content **(**Fig. 6A**)**. Triglyceride (TG) content was significantly lower in the MSC-CM group compared to the control group. The free cholesterol (FC) content tended to decrease in the MSC-CM group compared to controls, although the difference was not significant. The content of total cholesterol (TC) and phospholipid (PL) showed no significant difference. Next, PAS staining was performed to assess glycogen storage in liver tissue. There was no significant difference in the PAS-positive area between groups (Fig. 6B, C). To explore energetic status of the liver, hepatic content of ATP, β-hydroxybutyrate and Acetyl CoA at 168 h after PHx were measured. ATP and β-hydroxybutyrate content were significantly higher in the MSC-CM group compared to controls (Fig. 6D, E). In contrast, the MSC-CM group exhibited a significant decrease in acetyl CoA levels compared to the control group (Fig. 6F).

## Discussion

Rats fed the MCD diet were used as a model of MASLD. The MCD diet induces systemic lipodystrophy that increases influx of free fatty acids to the liver*,* resulting in oxidative stress and hepatocyte apoptosis. Glucose storage was significantly reduced compared to standard diet-fed mice [22]. Here, mice with MASLD induced by a methionine-choline deficient diet had poorer post-surgical outcomes following hepatectomy compared to their healthier counterparts [23], reflecting the increased complications and death rates observed in MASLD patients undergoing partial hepatic resection [24].

In our study, administration of hydrogel-encapsulated MSC-CM to the intra-mesenteric space in rodent MASLD models significantly promoted liver regeneration after partial hepatectomy even until the termination stage of liver regeneration. Additionally, the mode of regeneration was dominated by cellular hypertrophy with a suppression of cell division. Furthermore, this regeneration was accompanied by maintenance of mitochondrial function, such as ATP production and β-oxidation of fatty acids, so energy produced in the mitochondria seemingly fueled liver regeneration.

Hepatocyte hypertrophic regeneration after partial hepatectomy has been thought to occur only when proliferative potential of hepatocytes is compromised, such as hepatocytes deficient in specific genes related to the cell cycle [25], bleeding [26] during hepatectomy, or administration of dexamethasone [27]. Interestingly, Miyaoka et al. reported that hypertrophy and proliferation almost equally contribute to liver regeneration after 70% partial hepatectomy in normal murine liver [28], which is contrary to proliferation dominantly contributing to regeneration.

Here, expression of cell cycle entry marker PCNA increased in the MSC-CM group compared to controls. In parallel, cyclin D1 expression and mitosis were depressed in the MSC-CM group. The rate of Ki-67-positive nuclei was comparable between groups, suggesting no difference in proliferation. Concurrently, the liver regeneration rate was significantly increased in the MSC-CM group compared to controls at 30 h, which correspond to the progression/maintenance phase after hepatectomy. The above results indicate that MSC-CM-induced cell cycle arrest in fact *promoted* liver regeneration in a hepatocyte hypertrophic manner instead of hindering it.

Cytokine array analysis revealed that MSC-CM used in this study contained substantial amount of Activin-A. Activin-A plays the most crucial role in maintaining the homeostasis of liver weight. In a normal liver of quiescent state, moderate expression of Activin-A is observed. However, following partial hepatectomy, its expression dramatically decreases immediately. As regeneration progresses, its expression significantly increases, reaching its peak around when liver weight is restored to its original level [29]. Hepatocytes are the primary cells responsible for synthesis of Activin-A and exhibit a strong expression of type I and II activin receptors. Activin-A orchestrates inhibition of hepatocyte expansion via autocrine/paracrine pathways, highlighted by alterations in the expression of genes governing proliferation and senescence, a reduced propensity for cell division [30].

Moreover, functional analysis identified that hepatocyte nuclear factor (HNF)-4α might be critical targets of activin A signaling, implicating it in the regulation of hepatocyte proliferation and lipid homeostasis. Meanwhile activin-HNF4α-coagulation axis plays a crucial role in determining clinical outcomes of severe acute liver failure [31]. Thus, Activin-A does not simply inhibit proliferation of hepatocytes but also promotes their survival and affects lipid metabolism, which aligns with this study’s findings. To the best of our knowledge, effect of exogenous Activin-A administration after liver resection has not been reported previously. This unique pattern of hepatocyte hypertrophic regeneration demonstrated here through MSC-CM might be partially attributed to the action of Activin-A.

Previous research reported that impairments in oxidative phosphorylation such as decreased liver ATP synthesis increased production of ROS in MASH patients [32]. Therefore, mitochondrial dysfunction should play a significant role in the progression of MASH. Acetyl-CoA is normally generated through sugar and fatty acids (β-oxidation) metabolism and converts to ketone bodies in the liver under metabolic disturbances. Since acetyl-CoA produces ATP in the tricarboxylic acid (TCA) cycle, energy shortage occurs due to defective utilization of acetyl-CoA in starvation state and thereby enhancement of β-oxidation to generate ketone bodies is required. Intrahepatic glycogen and free glucose storage were significantly reduced in mice fed the MCD diet [33], indicating that supply shortage and impaired utilization of acetyl-CoA coexist in this animal model of MCD diet-induced MASLD-like features in the context of leanness, leading to ATP deficit in liver.

Analogous to our results, reduction in phosphatase and tensin homolog (PTEN) expression following hepatectomy enhances utilization of transient regeneration-associated steatosis (TRAS)-derived accumulated liver lipids by β-oxidation in mitochondria [34], providing energy for hypertrophic liver regeneration. Our results demonstrated that MSC-CM treatment improved liver regeneration rate as well as increased ATP and β-hydroxybutyrate content and decreased acetyl CoA and triglyceride in remnant liver until the termination phase of regeneration after hepatectomy. This implies that MSC-CM promoted liver regeneration not only in liver volume, but also in functionality by affecting lipid metabolism as fuel sources to support regeneration. Further studies are needed to clarify the functional link among MSC-CM, hepatocyte hypertrophic regeneration and β-oxidation.

This study also showed that the serum levels of IL-10 at 9 h and 30 h after hepatectomy were significantly higher in the MSC-CM group. IL-10 is a cytokine with anti-inflammatory properties that plays a pivotal role in modulating immune responses. It is produced by almost all subsets of leukocytes, including dendritic cells, macrophages, T cells, natural killer cells, B cells [35] and MSCs. IL-10 secreted by transplanted MSCs activated the mammalian target of rapamycin (mTOR) pathway and thereby enhanced mitochondrial function as well as correct abnormal lipid metabolism in treating post-hepatectomy liver failure [36]. Although MSC-CM used in our model contained IL-10, its concentration was not high compared to serum IL-10 levels. It is possible that the other factors in MSC-CM acted on IL-10 producing cells to enhance IL-10 production.

Olfactory receptors (ORs) are seven-transmembrane G protein-coupled receptors (GPCRs) located mainly in olfactory sensory neurons of the olfactory epithelium. GCPRs play a crucial role as chemosensors by detecting and decoding chemical signals in their environment [37]. ORs are ectopically expressed throughout the body including liver [38], heart [39], kidney [40], and pancreas [41]. The liver is the largest metabolic organ regulating whole-body homeostasis by sensing and detoxifying drugs, alcohol, and environmental toxins, metabolizing nutrients, synthesizing and secreting bile acids, and removing toxins and other waste products from the blood. Thus, the liver appears to be primed to utilize these ORs.

Recently, several groups have identified distinctive metabolic functions for hepatic ORs. *Olfr734* responds to Asprosin and serves as a fasting-induced glucogenic hormone [42]. Asprosin is encoded by *FBN1* gene whose mRNA expression is confirmed to be abundant in MSCs. In addition, *Olfr544* can trigger lipolysis upon activation in diabetic mice [43]. Notably, GO enrichment analysis showed that expression of functional category sensory perception of smell genes were enhanced following hepatectomy (the control group) compared with the MCD-fed alone group, whereas those genes were suppressed in the MSC-CM group compared with the control group. For the brain to identify a wide variety of odors, it is crucial that each sensor carries only one type of olfactory receptor, a principle known as the 'one sensor, one receptor' rule. Once a specific set of ORs is activated, inhibiting activation of other OR genes in that cell becomes necessary. This is ensured by a negative feedback mechanism deactivating additional specific control regions known as locus control regions and their associated protein factors, thereby upholding the principle of one neuron per receptor [44].

This led us to speculate that MSC-CM might contain factors serving as ligands for certain olfactory receptors, and their delivery subsequently suppressed expression of other olfactory gene receptors. ORs in the liver could potentially function as chemical sensors, altering liver metabolism. Further detailed research is necessary to determine whether MSC-CM contains ligands for specific ORs, and the impact of activating those ORs on liver regeneration.

Pharmacological alteration of macrophage polarization towards an M2 phenotype has been shown to partially reverse liver steatosis [45]. Moreover, in animal models of acute liver failure (ALF), MCP-1 and Siglec-9 secreted from human exfoliated deciduous tooth-derived mesenchymal stem cells have been shown synergistically to promote the M2 differentiation of bone marrow-derived macrophages via CCR2 and thereby improved the survival of ALF rats [46]. In our model, MSC-CM containing certain amounts of MCP-1 did not alter the number of CD163-positive cells, a specific marker of M2-type macrophage, implying that regeneration effect of MSC-CM was not M2-type macrophage-mediated.

In liver regeneration after partial hepatectomy, vascular remodeling through autocrine and paracrine control mediated by angiogenic growth factors and their receptors is known to be involved. Hepatocyte produce VEGF within 48 h-72 h after PHx, promoting sinusoidal endothelial cell proliferation and hepatocyte proliferation [47]. Additionally, VEGF promotes hepatocyte proliferation by inducing HGF production through VEGFR1 in LSECs [48]. The MSC-CM here contained certain amount of VEGF, so we assessed angiogenesis during regeneration by CD31 immunohistochemistry. Unexpectedly, results revealed that MSC-CM did not seem to affect angiogenesis in this model.

Various differences in liver metabolic functions between humans and experimental animals such as mice and rats have been reported. Recent studies have shown that human liver organoids can replicate inflammation and fibrosis in MASLD [49, 50]. To determine whether our findings can be applied to human clinical practice, future research should investigate the effects of human MSC-CM on post-hepatectomy liver regeneration with MASLD as background liver disease in an in vitro model using human liver organoids.

## Conclusions

Delivering MSC-CM encapsulated hydrogel to intra-mesenteric space in a controlled-release manner during hepatectomy for MASLD patients might be a feasible and safe approach, potentially serving as an attractive therapeutic modality that supports the reduction of post-surgical complications and death rates.

## Supplementary Information

Additional file1

## Data Availability

All data generated or analyzed during this study were included in this published article and its supplementary information files.

## Abbreviations

7-AAD: 7-Amino-actinomycin D
ALT: Alanine transaminase
ATP: Adenosine triphosphate
DAVID: Database for annotation, visualization and integrated discovery
FAO: Fatty acid beta-oxidation
FC: Free cholesterol
GO: Gene ontology
GPCRs: G protein-coupled receptors
HNF-4α: Hepatocyte nuclear factor-4α
IFN-γ: Interferon-γ
MASH: Metabolic dysfunction-associated steatohepatitis
MASLD: Metabolic dysfunction-associated steatotic liver disease
MCD: Methionine and choline–deficient
MCP-1: Monocyte chemoattractant protein-1
MSCs: Mesenchymal stem cells
MSC-CM: MSCs conditioned medium
mTOR: Mammalian target of rapamycin
ORs: Olfactory receptors
PAS: Periodic acid-Schiff
PCNA: Proliferating cell nuclear antigen
PHx: Partial hepatectomy
PL: Phospholipid
PTEN: Phosphatase and tensin homolog
ROS: Reactive oxygen species
SECs: Sinusoidal endothelial cells
TC: Total cholesterol
TCA: Tricarboxylic acid
TG: Triglyceride
TNF-α: Tumor necrosis factor-α
TRAS: Transient regeneration-associated steatosis
TUNEL: TdT-mediated dUTP Nick-end labeling
