# Supplementary material for: Controlled release of hydrogel-encapsulated mesenchymal stem cells-conditioned medium promotes functional liver regeneration after hepatectomy in metabolic dysfunction-associated steatotic liver disease
Source: Stem Cell Res Ther. 2024 Nov 4;15:395. doi: 10.1186/s13287-024-03993-w (PMC11536549; doi:10.1186/s13287-024-03993-w)
Supplement: Supplementary file 1 — Additional file1 [file 13287_2024_3993_MOESM1_ESM.docx]

**Supplementary Figure S1.**

**A**


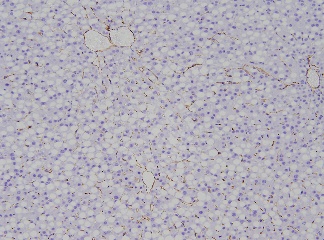

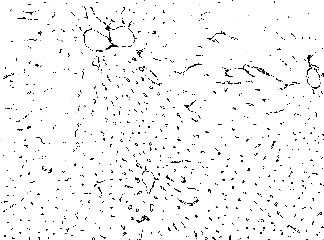


Control


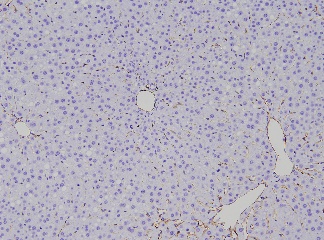


MSC-CM


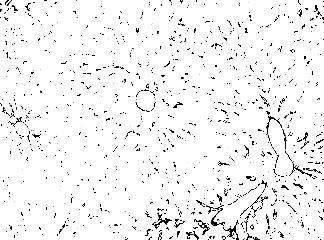


**CD31 positive area**

**B**

(A) Representative liver sections stained by CD31 at 30h after hepatectomy.

(B) Quantification of CD31 positive area at 30h after hepatectomy (n = 4-5/group).

**Supplementary Figure S2.**

**A**


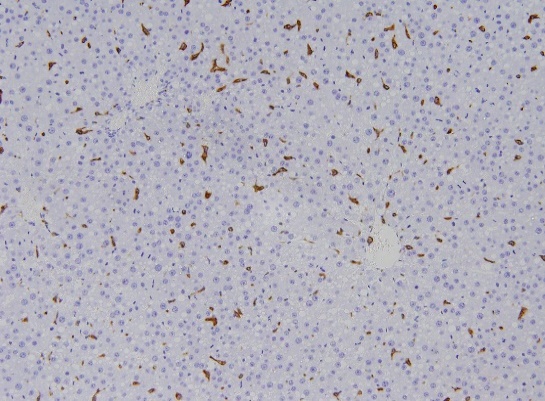


Control

**CD163-positive cell**


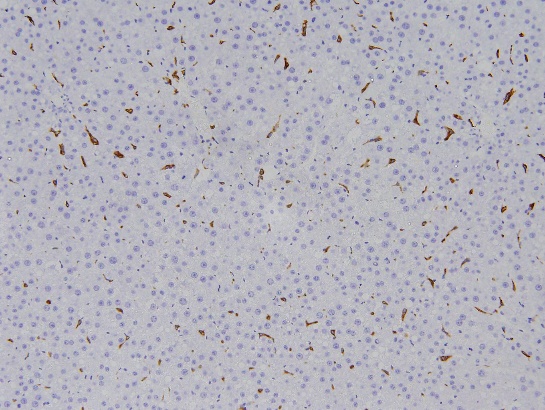


MSC-CM

**B**

(A) Representative liver sections stained by CD163 at 30h after hepatectomy.

(B) Quantification of CD163-positive nuclei (%) at 30h after hepatectomy (n = 4-5/group).

**Supplementary Figure S3.**


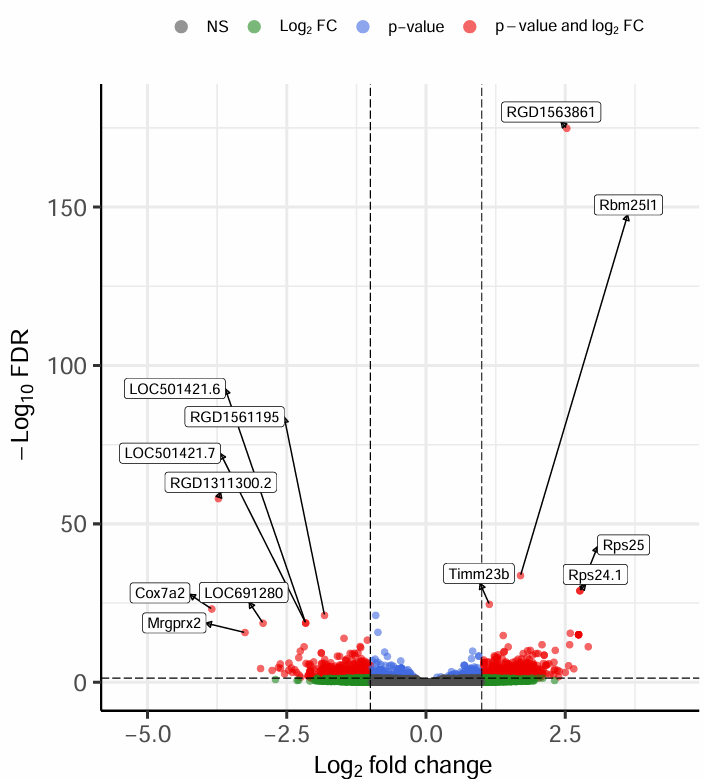


Differential expression analysis of liver tissue mRNA expression at 9h for control group versus MSC-CM group. Volcano plots showing all the DEGs.

**Supplementary Table S1.**


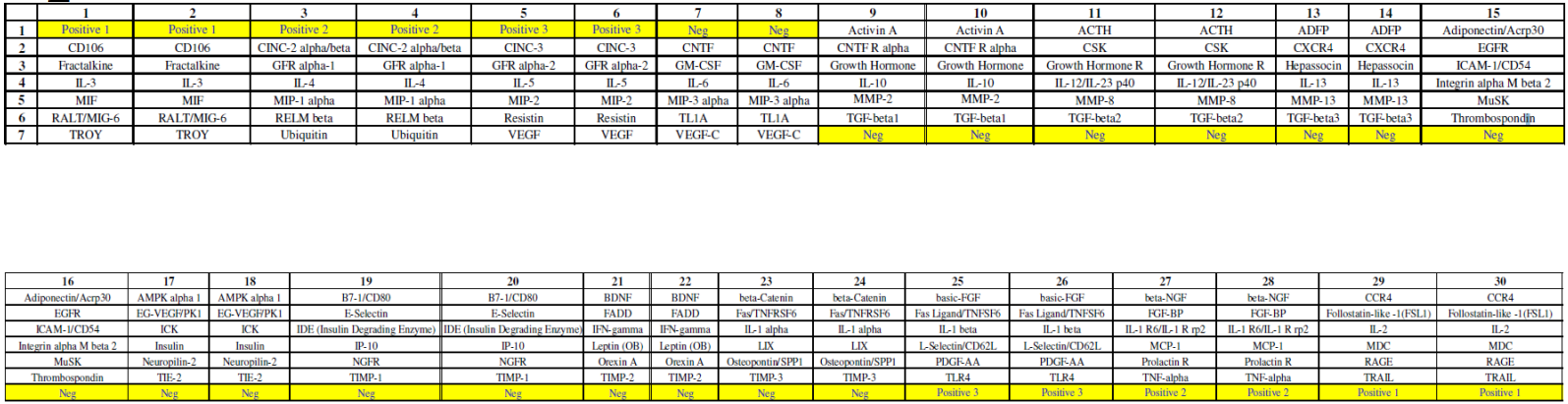


**Supplementary Table S2.** Differential gene expression analysis of Standard diet group versus MCD diet group. Top 10 DEGs in ascending order of FDRs.

| **Symbol** | **logFC** | **logSIGNAL** | ***P* value** | **FDR** |
| --- | --- | --- | --- | --- |
| LOC100360095 | -7.44 | 9.87 | 0 | 0 |
| Cyp7a1 | 5.08 | 8.76 | 0 | 0 |
| Igfbp1 | 4.15 | 9.09 | 0 | 0 |
| Mt1m | -1.74 | 10.0 | 0 | 0 |
| Hamp | 1.19 | 10.8 | 6.51E-319 | 2.94E-315 |
| Cyp2c11 | -1.67 | 9.70 | 1.20E-276 | 4.53E-273 |
| Mt1a.1 | -1.53 | 9.88 | 1.54E-264 | 4.96E-261 |
| Hsd17b2 | -2.67 | 8.69 | 2.19E-254 | 6.18E-251 |
| Dhrs7 | -4.00 | 7.95 | 3.13E-240 | 7.87E-237 |
| G6pc | 2.03 | 9.21 | 2.29E-239 | 5.17E-236 |

**Supplementary Table S3.** Differential gene expression analysis of MCD diet group versus control group. Top 10 DEGs in ascending order of FDRs.

| **Symbol** | **logFC** | **logSIGNAL** | ***P* value** | **FDR** |
| --- | --- | --- | --- | --- |
| Mt2A | 5.88 | 10.2 | 1.14E-64 | 2.58E-60 |
| Mir770 | 5.98 | 9.50 | 2.49E-62 | 2.82E-58 |
| Cyp7a1 | -6.24 | 8.73 | 8.13E-52 | 6.13E-48 |
| Fads1 | -5.20 | 9.20 | 1.23E-47 | 6.94E-44 |
| Rnase4 | -5.16 | 9.00 | 6.29E-46 | 2.65E-42 |
| Hmgcs1 | -5.11 | 9.09 | 7.03E-46 | 2.65E-42 |
| Cth | -4.87 | 9.33 | 6.16E-45 | 1.99E-41 |
| Cyp3a23.3a1.1 | -6.14 | 7.98 | 1.05E-43 | 2.97E-40 |
| Vom2r70 | 5.90 | 7.08 | 7.68E-42 | 1.93E-38 |
| Pkib.1 | 4.52 | 8.78 | 8.57E-42 | 1.94E-38 |

**Supplementary Table S4.** GO Biological Processes that are enriched in DEGs in Standard diet group versus MCD diet group. Top 10 GO biological processes in ascending order of *P* values.

| **GO.ID** | **Term** | **Annotated** | **Significant** | **Expected** | ***P* value** |
| --- | --- | --- | --- | --- | --- |
| GO:0044281 | small molecule metabolic process | 1620 | 99 | 39.4 | 1.30E-18 |
| GO:0043436 | oxoacid metabolic process | 900 | 66 | 21.9 | 4.50E-16 |
| GO:0006082 | organic acid metabolic process | 910 | 66 | 22.1 | 7.80E-16 |
| GO:0006629 | lipid metabolic process | 1303 | 81 | 31.7 | 1.70E-15 |
| GO:0019752 | carboxylic acid metabolic process | 887 | 63 | 21.6 | 1.10E-14 |
| GO:0008202 | steroid metabolic process | 310 | 35 | 7.56 | 3.50E-14 |
| GO:0009410 | response to xenobiotic stimulus | 679 | 53 | 16.5 | 4.40E-14 |
| GO:0014070 | response to organic cyclic compound | 1386 | 79 | 33.7 | 4.30E-13 |
| GO:0032787 | monocarboxylic acid metabolic process | 626 | 48 | 15.2 | 1.50E-12 |
| GO:0044283 | small molecule biosynthetic process | 536 | 43 | 13.0 | 5.70E-12 |

**Supplementary Table S5.** GO Biological Processes that are enriched in DEGs in MCD diet group versus control group. Top 10 GO biological processes in ascending order of *P* values.

| **GO.ID** | **Term** | **Annotated** | **Significant** | **Expected** | **Fisher’s Test** |
| --- | --- | --- | --- | --- | --- |
| GO:0050877 | nervous system process | 1351 | 1133 | 921 | < 1e-30 |
| GO:0007186 | G protein-coupled receptor signaling pathway | 981 | 839 | 668 | < 1e-30 |
| GO:0007600 | sensory perception | 755 | 663 | 514 | < 1e-30 |
| GO:0003008 | system process | 2031 | 1612 | 1384 | < 1e-30 |
| GO:0032501 | multicellular organismal process | 7090 | 5148 | 4833 | 1.40E-28 |
| GO:0007606 | sensory perception of chemical stimulus | 262 | 249 | 178 | 2.20E-27 |
| GO:0051606 | detection of stimulus | 311 | 276 | 212 | 8.00E-18 |
| GO:0050906 | detection of stimulus involved in sensory perception | 219 | 201 | 149 | 3.40E-17 |
| GO:0007268 | chemical synaptic transmission | 864 | 695 | 589 | 7.00E-17 |
| GO:0098916 | anterograde trans-synaptic signaling | 864 | 695 | 589 | 7.00E-17 |
